# Potential Role of Honey in Learning and Memory

**DOI:** 10.3390/medsci3020003

**Published:** 2015-04-09

**Authors:** Zahiruddin Othman, Rahimah Zakaria, Nik Hazlina Nik Hussain, Asma’ Hassan, Nazlahshaniza Shafin, Badriya Al-Rahbi, Asma Hayati Ahmad

**Affiliations:** 1 Department of Psychiatry, School of Medical Sciences, Universiti Sains Malaysia, 16150 Kubang Kerian, Malaysia; 2 Department of Physiology, School of Medical Sciences, Universiti Sains Malaysia, 16150 Kubang Kerian, Malaysia; E-Mails: rahimah@usm.my (R.Z.); drshaniza@usm.my (N.S.); asmakck@usm.my (A.H.A.); 3 Women’s Health Development Unit, School of Medical Sciences, Universiti Sains Malaysia, 16150 Kubang Kerian, Malaysia; E-Mail: hazlinakck@usm.my; 4 Faculty of Medicine, Universiti Sultan Zainal Abidin (UniSZA), Jalan Sultan Mahmud, 20400 Kuala Terengganu, Malaysia; E-Mail: asmahassan@unisza.edu.my; 5 Institute of Health Sciences, Muscat P.O. Box 3720, Ruwi Code 112, Oman; E-Mail: bidoor94@hotmail.com

**Keywords:** Tualang honey, learning, memory, BDNF, ACh, AChE

## Abstract

The composition and physicochemical properties of honey are variable depending on its floral source and often named according to the geographical location. The potential medicinal benefits of Tualang honey, a multifloral jungle honey found in Malaysia, have recently been attracting attention because of its reported beneficial effects in various diseases. This paper reviews the effects of honey, particularly Tualang honey, on learning and memory. Information regarding the effects of Tualang honey on learning and memory in human as well as animal models is gleaned to hypothesize its underlying mechanisms. These studies show that Tualang honey improves morphology of memory-related brain areas, reduces brain oxidative stress, increases brain-derived neurotrophic factor (BDNF) and *acetylcholine* (ACh) concentrations, and reduces *acetylcholinesterase* (AChE) in the brain homogenates. Its anti-inflammatory roles in reducing inflammatory trigger and microglial activation have yet to be investigated. It is hypothesized that the improvement in learning and memory following Tualang honey supplementation is due to the significant improvement in brain morphology and enhancement of brain cholinergic system secondary to reduction in brain oxidative damage and/or upregulation of BDNF concentration. Further studies are imperative to elucidate the molecular mechanism of actions.

## 1. Introduction

Tualang honey derives its name from the tualang tree (*Koompassia excelsa*). The *Koompassia excelsa* tree is among the tallest trees in the world, and also one of the most prominent trees in the tropical rainforests of the Sunda Shelf. The grey, whitish bark of the tree, large bole, and often handsome crown makes it stand out amongst the other trees. It can reach up to 250 feet (approximately 80 m) in height and is found in the tropical rain forests of Sumatra, Borneo, South Thailand, and Peninsular Malaysia [1]. It is known by different names in different regions—*Mengaris* in Brunei and Sabah, *Tualang* in Peninsular Malaysia, *Sialang* in Indonesia, and *Tapang* in Sarawak. The name tualang comes from the Malay words *tua* which means old, and *helang* which means eagle. Immense parabolic honey combs hang from the bottom of their branches. The combs are up to 6 feet across and each may contain as many as 30,000 bees. One tualang tree can contain more than 100 nests and can yield about 450 kg of honey [1,2].

Tualang honey is produced by rock bee (*Apis dorsata*). It has a dark brown appearance, a pH of 3.55–4.00 and a specific gravity of 1.335 [3]. It is more acidic than other local Malaysian honeys, such as Kelulut Hitam, Kelulut Putih, and Gelam [3]. This characteristic makes Tualang honey effective against several pathogenic microorganisms [3,4]. The concentration of 5-(hydroxymethyl) furfural (HMF) in Tualang honey is greater than in other Malaysian honeys [5].

Tualang honey contains more phenolic acids and flavonoids than Manuka honey and other local Malaysian honeys [6]. A total of six phenolic acids (gallic, syringic, benzoic, trans-cinnamic, p-coumaric, and caffeic acids) and five flavonoids (catechin, kaempferol, naringenin, luteolin and apigenin) are found in Tualang honey [6,7,8]. Hydrocarbons constitute more than half (58.5%) of its composition. These include alcohols, ketones, aldehydes, furans, terpenes, flavonoids, and phenols [6]. Some compounds found in Tualang honey previously not reported in other honeys are stearic acids, 2-cyclopentene-1,4,-dione, 2[3H]-furanone or dihydro-butyrolactone, gamma-crotonolactone or 2[5H]-furanone, 2-hydroxy-2-cyclopenten-1-one, hyacinthin, 2,4-dihydroxy-2,5-dimethyl-3[2H]-furan-3-one, and phenylethanol [6,8,9]. The vitamins, enzymes, amino acids, trace elements, and other compounds in Tualang honey have yet to be quantified.

## 2. Honey in Learning and Memory—Evidence from Human Studies

Within the last decade, studies have been conducted using regional honey to investigate effects on learning and memory. Al-Himyari *et al.* [10] conducted a five-year pilot study involving 2290 cognitively intact subjects and 603 with mild cognitive impairment aged 65 and older. They were randomized to receive either one daily tablespoon of Middle East honey or placebo. They found that only 95 subjects who received honey compared to 394 who received placebo developed dementia. The study concluded that honey and its properties act as natural preventive therapies for both cognitive decline and dementia.

Recent studies show that learning and memory is affected by a loss of estrogen (as in a case of postmenopausal women or ovariectomized rats), indicating a causal relationship between estrogen reduction and decrease in cognitive function [11,12,13,14]. Following this, a study using Tualang honey was conducted on 102 healthy postmenopausal women [15] randomly assigned to one of three groups; untreated control, estrogen plus progestin therapy and Tualang honey. The participants in Tualang honey and estrogen plus progestin therapy groups received 20 g Tualang honey supplement and Femoston conti 1/5 (1 mg 17-β estradiol and 5 mg dydrogesterone), respectively, daily for 16 weeks. The participants’ memory and oxidative stress status were assessed pre- and post-intervention. Postmenopausal women who received Tualang honey showed improvement in their immediate memory but not in immediate memory after interference and delayed recall. This result was comparable with the memory improvement seen in women receiving estrogen plus progestin therapy [15].

Honey was also used in combination with other herbal preparations to investigate the effects of electroconvulsive therapy (ECT) on memory. A randomized double-blind clinical trial was conducted on 74 patients with mood disorders who were candidates for ECT. The patients were given 9 g of a herbal combination comprising *Crocus sativus*, *Cyperus rotundus* and honey (*n* = 36), or a placebo (*n* = 38) twice daily for 40 days from the ECT initiation time. Both the placebo and herbal combination capsules were supplied by a pharmacological institute in Shahrekord, Iran. ECT was performed three times a week and cognitive status was examined using Addenbrooke's Cognitive Examination-Revised (ACE-R) test on five occasions, *i.e.*, before ECT, after the fourth and the last ECT sessions, and one and two months following the last session. ACE-R scores increased significantly in the intervention group one and two months following the last ECT session. The findings confirmed the memory improving effects of *Crocus sativus*, *Cyperus rotundus* and honey [16]. However, data on the effect of each component of the herbal combination was not available to confirm the contributing effect of honey.

## 3. Honey in Learning and Memory—Evidence from Animal Studies

A few studies also looked at the effects of honey in animals. A study conducted by Chepulis *et al.* (2009) found better improvement in spatial memory and reduction in anxiety in rodents fed with beech forest honeydew honey compared to rodents fed with sucrose or sugar-free diet over a 12-month trial period [17]. They concluded that early introduction of honey diet is beneficial and can improve memory loss and cognitive decline associated with aging. While it is possible that the learning and memory improvement following honey supplementation is due to the sugar content in honey, these investigators controlled for this effect using sucrose.

In another study by Akanmu and collegues [18], the neurological effects of Nigerian honey were investigated by assessing its (1) spatial working memory in mice using the Y-maze test and pentobarbital-induced hypnosis and assessment; (2) anxiolytic activities using hole-board and elevated plus maze tests; (3) anticonvulsant activity in a picrotoxin seizure model; (4) antinociceptive activity in hot-plate and tail-flick tests; and (5) antidepressant effects using the forced swimming test. The authors of that study concluded that honey is a functional food that improves spatial working memory and possesses anxiolytic, antinociceptive, anticonvulsant, and antidepressant effects.

Honey in herbal preparation was used by Cai *et al.* [19] in an animal study. They reported that Kyung-Ok-Ko (KOK), a traditional herbal prescription composed of *Rehmannia glutinosa var. purpurae*, *Panax ginseng*, *Poria cocos*, *Lycium chinense*, *Aquillaria agallocha* and honey (0.25, 0.5, 1, or 2 g/kg) significantly attenuated ischemia-induced cognitive impairments in gerbils. KOK (Lot No., OV30) standardized with 5-hydroxymethyl furaldehyde (9.4%) for consistency of quality was donated by Kwang Dong Pharmaceutical Co. (Pyongtaek, Korea). The findings suggested that the neuroprotective effects of KOK may be mediated by its anti-inflammatory activities, resulting in the attenuation of memory impairment [19]. This study however did not identify the specific role of honey in neuroprotection.

Most of the previous animal studies on the cognitive effects of honey were conducted in young animals. A recent animal study by Al-Rahbi and colleagues administered Tualang honey to 40 ovariectomised (OVX) and 20 sham-operated female Sprague-Dawley rats. The rats’ memory performance was assessed using Novel Object Recognition test. Tualang honey treatment improved both short-term and long-term memory, and enhanced the neuronal proliferation of hippocampal CA2, CA3 and DG regions compared to untreated groups [20].

Recently, it was documented that pretreatment with *Swarnabhasma* (gold) mixed with honey (called *Swarnaprashana)* for 15 days in young and aged Swiss albino mice exhibited significant improvement in learning and memory and significant decrease in whole brain acetylcholinesterase (AChE) activity [21]. However, the specific effect of honey in the preparation was not mentioned in this study.

## 4. Possible Mechanisms of Tualang Honey in Learning and Memory

### 4.1. Oxidative Stress and Tualang Honey

The brain has one of the highest respiratory rates and generates oxidative damage that progressively increases over time [22]. Neurons are particularly vulnerable to cumulative oxidative damage because they are non-dividing cells that survive for decades. The generation of oxidants damages proteins, lipids, and nucleotides, possibly contributing significantly to neuron dysfunction and degeneration associated with ageing and neurodegenerative diseases [23].

Cognitive impairment can be prevented or significantly delayed by the increased intake of antioxidants in the diet, such as vitamins C and E, and β-carotene [24,25]. Biochemical and physiological studies implicate oxidative damage to central nervous system microvasculature in the aetiology of neurodegenerative diseases; including Alzheimer’s disease [26]. An epidemiological and correlational study has demonstrated significant relationships between cognitive impairment and low vitamin C intake and plasma ascorbic acid levels [27]. In another study, Ortega *et al.* [28] found a significant relationship between cognitive function of elderly people and 10 dietary intake variables, including non-enzymatic antioxidants, such as vitamin E, β-carotene, and folate. A cross-sectional study involving 97 healthy Caucasian postmenopausal women found a positive association between antioxidant intake and cognition score [29]. A recent study by Devore *et al.* [30] indicated that long-term antioxidant intake e.g., carotenoids, has cognitive benefits in older adults.

Over the past few years, there has been a surge of interest in the ability of honey as an antioxidant followed by its protective effects on brain and other organs. Tualang honey, in particular, has been shown to exhibit good antioxidant and antiradical activities [6]. A study conducted on postmenopausal women showed that plasma glutathione peroxidase (GPx) and catalase (CAT) activities were notably increased, and plasma 4-hydroxynonenal (4-HNE) level significantly decreased in the women who received estrogen progestin therapy and Tualang honey supplementation. Tualang honey supplementation for 16 weeks was able to reduce blood oxidative stress levels/activities of postmenopausal women comparable to those who received estrogen progestin therapy [31]. Based on the above findings, it can be concluded that the elevation in plasma antioxidant enzymes activities and reduction in plasma lipid peroxidation may reflect similar changes in the memory-related brain areas such as hippocampus and prefrontal cortex [20]. This was clinically supported by the improvement in memory functions of postmenopausal women who received either estrogen progestin or Tualang honey [15].

Similar findings related to the influence of high radical scavenging activity of Tualang honey on cognitive functions were noted in an animal study performed by Al-Rahbi *et al.* [32]. The study showed that daily consumption of Tualang honey for 18 days in female ovariectomized rats is able to increase superoxide dismutase (SOD), glutathione *S*-transferase (GST), GPx, and glutathione reductase (GR) activities, and decreases malondialdehyde (MDA) and protein carbonyl (PCO) levels in the brain homogenate of Tualang honey-treated group as compared with the untreated stressed OVX rats. These findings are in line with the study by Oyefuga and colleagues [33] who showed that both short and long term (short term—3 weeks and long term—12 weeks) honey supplementations at a dose of 250 mg/kg body weight may increase the brain protein and CAT activities of brain cells, suggesting a significant increase in antioxidant capacity thus augmenting defenses against oxidative cell damage, cell injury, and the degenerative process of cell components *i.e.*, mitochondria, microsomes and DNA.

The positive effect on rat brain is probably partly due to the antioxidant properties found in Tualang honey such as flavonoids (catechin, kaempferol, naringenin, luteolin and apigenin) and phenolic acids (gallic, syringic, benzoic, trans-cinnamic, p-coumaric, and caffeic acids) [7,8,9]. The memory-ameliorating effects of gallic acid found in honey were confirmed by Al Mansouri *et al.* [34], by revealing its neuroprotective effect on 6-hydroxydopamine-induced and cerebral oxidative stress-induced memory deficits. Gallic acid improved memory concomitant with increases in the total thiol pool and GPx activity and decreased MDA in the hippocampus and striatum [35].

In a rat model of Alzheimer’s disease, the administration of naringenin reverses the learning, memory, and cognitive impairments caused by intracerebroventricular administration of streptozotocin [36]. Treatment with naringenin also increases the pool of reduced glutathione (GSH) and the activities of GPx, GR, GST and SOD in the hippocampus in another rat model of Alzheimer’s disease [37].

Previous studies also demonstrated the oxidative stress reduction capacity of p-coumaric acid, kaempferol, luteolin and apigenin which are found in honey [38]. P-coumaric acid is able to increase the levels of GSH, SOD, and CAT activities with concomitant reduction of MDA in doxorubicin-induced cardiotoxicity [39]. The administration of kaempferol has been reported to increase SOD and GPx activities, reduce MDA in the substantia nigra and prevent neuronal loss induced by MPTP (1-methyl-4-phenyl-1,2,3,6-tetrahydropyridine) [40]. The augmenting effect of luteolin on Mn-SOD and (Cu/Zn)-SOD activities as well as on the GSH levels in the cortex and hippocampus is associated with the amelioration of amyloid beta (1–40)-induced oxidative stress and cognitive deficits [41]. Apigenin inhibits the kainic acid-induced excitotoxicity of hippocampal cells in a dose-dependent manner by quenching reactive oxygen species and by inhibiting the depletion of GSH levels [42].

Tualang honey consumption enhances the defense mechanism against oxidative stress and attenuates free radical-mediated molecular destruction leading to improved arrangement and number of Nissl positive cells in the mPFC and hippocampal neurons [19,32,43]. Thus, it can be suggested that Tualang honey reduces oxidative damage and stimulates neurogenesis that underlies learning and memory performance in rats.

### 4.2. Cholinergic System and Tualang Honey

The functional decline of memory is partially attributed to defects in cholinergic transmission. Evidence from a previous animal study noted a large decrease in the rates of spontaneous or evoked release of acetylcholine (ACh) in the aging cerebrum as compared to non-ageing rats indicating that the functional defect in the cholinergic transmission of the aging cerebrum is possibly due to a defective release mechanism of this transmitter [44]. Clinical studies suggest that the marked cognitive impairment seen in Alzheimer patients results from impaired cholinergic neurotransmission due to selective damage of specific neuronal circuits in the neocortex, hippocampus, and basal forebrain cholinergic system [45].

In addition, it has been reported that cholinergic neurotransmission is gradually impaired in Alzheimer’s disease, as demonstrated by a marked loss of cholinergic neurons and substantial reductions in the numbers of nicotinic receptors in the brain [46]. In Alzheimer’s disease, the decrease in neocortical choline acetyltransferase (ChAT) is correlated with the number of neurons in the nucleus of Meynert suggesting that primary degeneration of these cholinergic neurons may be related, directly or indirectly, to declining cognitive function [47,48].

Findings from the study of Al-Rahbi *et al.* [43] showed reduced concentrations of ACh and increased AChE in the brain homogenates of stressed OVX rats compared with nonstressed sham-operated controls and the effects were reversed after treatment with Tualang honey. Honey has been reported to contain choline, ACh [49], naringenin and chlorogenic acid [7,8,9].

Treatment with naringenin increases ChAT in the hippocampus in a rat model of Alzheimer’s disease- (AD-) type neurodegeneration with cognitive impairment (ADTNDCI), with a concomitant decrease in the loss of ChAT positive neurons and impairments in spatial learning and memory [37]. Chlorogenic acid is a derivative of caffeic acid and is another common phenolic acid that is found in honey. In a study by Kwon *et al.* [50], the neuroprotective effects of chlorogenic acid on scopolamine-induced learning and memory impairment were investigated using several behavioral tests, such as the Y-maze, passive avoidance, and Morris water maze tests. Chlorogenic acid was found to significantly improve memory-related performance in all of the tests. It is concluded that chlorogenic acid may exert antiamnesic activity via the inhibition of AChE in the hippocampus and frontal cortex in both *ex vivo* and *in vitro* model systems [50].

### 4.3. BDNF and Tualang Honey

A growing body of evidence has emerged suggesting that brain-derived neurotrophic factor (BDNF) plays a crucial role in learning and memory [51,52] consistent with the view that those activity-dependent changes in synaptic strength underlie memory processing and storage [53]. BDNF is involved in the formation of different types of memories and is also critical for maintaining long-lasting storage of information in hippocampus, amygdala and insular cortex many hours after learning occurs [54]. BDNF may be relevant to counteract the natural process of memory decay, which is typical in aging and is exacerbated in some neurodegenerative disorders such as Huntington’s disease and Alzheimer’s disease. The conversion of short-term memory (STM) into long-term memory (LTM) is regulated at the molecular level in neurons [55,56,57] and involves the synthesis of new proteins that control neuronal morphology and connectivity [58]. A growing body of evidence indicates that BDNF plays a key role in the regulation of both short-term synaptic function and long-term activity-dependent synaptic plasticity during memory formation [59,60,61]. Decreased expression of BDNF contributes to hippocampal atrophy and neuronal loss in experimental animals [62].

BDNF concentration is significantly decreased in stressed OVX rats compared to other experimental groups and the concentration is restored to normal following Tualang honey treatment [63]. Since Tualang honey is a phytoestrogen rich in flavonoids, it is possible that the mechanisms of improved learning and memory are similar to other phytochemical food rich in flavonoids such as green tea, blueberry, and Ginkgo biloba which have been shown to increase hippocampal BDNF levels [64,65,66,67,68]. Recently, another study reported that flavonoids-induced synthesis and secretion of BDNF in cultured rat astrocytes are mediated by the estrogen receptor [69], providing further indication of the possible underlying mechanism and needing further evaluation.

### 4.4. Anti-Inflammatory and Tualang Honey

Ischemia-induced neuroinflammation by activating microglia, and neuroinflammatory processes in the brain are believed to play a crucial role in the development of neurodegenerative diseases as well as in neuronal injury associated with stroke [70,71]. Tualang honey has been shown to possess anti-inflammatory effects. It improves wound healing by abating the oedema, inflammation, and exudation that commonly occur in all types of wounds by stimulating growth of epithelial cells and fibroblasts [72,73,74]. Tualang honey also decreases the wound size of burns and provides enhanced control and containment of burn infections, especially by bacteria such as *Pseudomonas aeruginosa* [75]. In addition, it shows anti-inflammatory effect which is almost equal to the conventional treatment in treating alkali injury in rabbit’s eye [76]. However, its anti-inflammatory role in reducing the triggering of neuroinflammatory and microglial activation warrants further investigation.

**Figure 1:**
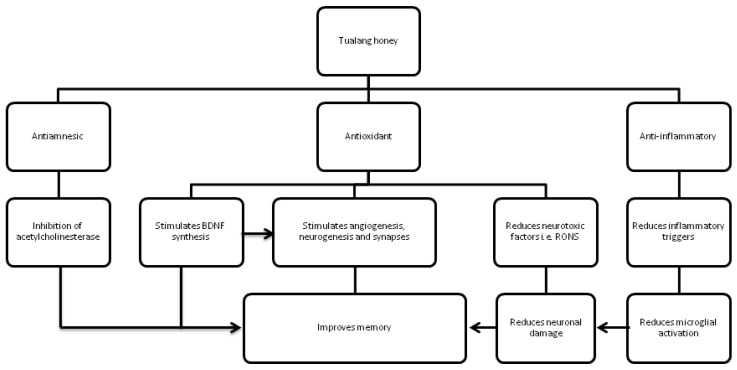
Proposed roles of Tualang honey in learning and memory.

To date, studies on the effects of Tualang honey on learning and memory have been conducted using the whole composition of Tualang honey. Limitations of these studies meant that bioactive compounds which provide the beneficial effect and their molecular mechanism of action could not be identified. The proposed roles of Tualang honey in learning and memory is shown in Figure 1.

## 5. Conclusions

The improvement of learning and memory by Tualang honey may be due to the significant improvement in brain morphology and enhancement of the brain cholinergic system, which is possibly secondary to reduction in brain oxidative damage and/or upregulation of BDNF concentration. Tualang honey also has the capacity to inhibit the triggering of neuroinflammatory and microglial activation. Future studies should focus on its extract with known bioactive compounds, its molecular mechanisms and its essential components acting to improve learning and memory.
